# Impacts of dimethyl phthalate on the bacterial community and functions in black soils

**DOI:** 10.3389/fmicb.2015.00405

**Published:** 2015-05-05

**Authors:** Zhi-Gang Wang, Yun-Long Hu, Wei-Hui Xu, Shuai Liu, Ying Hu, Ying Zhang

**Affiliations:** ^1^Department of Biotechnology, Institute of Life Science and Agriculture and Forestry, Qiqihar UniversityQiqihar, China; ^2^Department of Environmental Science and Engineering, Institute of Municipal Environment and Engineering, Harbin Industry UniversityHarbin, China; ^3^Department of Environmental Science, Institute of Resources and Environment, Northeast Agricultural UniversityHarbin, China

**Keywords:** bacterial community, metabolic function, black soils, dimethyl phthalate (DMP), contamination, biodiversity

## Abstract

Dimethyl phthalate (DMP), a known endocrine disruptor and one of the phthalate esters (PAEs), is a ubiquitous pollutant. Its impacts on living organisms have aroused great concern. In this study, the impacts of DMP contamination on bacterial communities and functions were tested by using microcosm model in black soils. The results showed that the operational taxonomic unit (OTUs) richness and bacterial diversity were reduced by DMP contamination. The relative percentages of some genera associated with nitrogen metabolism were increased by DMP contamination, while the relative percentages of some other genera that were extremely beneficial to soil health were decreased by DMP contamination. Further, the relative percentages of some genera that possessed the capability to degrade DMP were increased by the DMP treatment at low concentrations (5, 10, and 20 mg/kg), but were decreased by the high concentration DMP treatment (40 mg/kg). Clearly, DMP contamination changed the bacterial community structure and disturbed the metabolic activity and functional diversity of the microbes in black soils. Our results suggest that DMP pollution can alter the metabolism and biodiversity of black soil microorganisms, thereby directly impact fertility and ecosystem functions.

## Introduction

Dimethyl phthalate (DMP) is commonly used as a plasticizer to impart flexibility to rigid polyvinylchloride (PVC) resins (Boonnorat et al., 2014). As one of the phthalate esters (PAEs), DMP is the most extensively used compound for manufacturing various consumer products, and the consumption of it is increasing continually (Fang et al., 2009; Prasad and Suresh, 2015) every year. Because DMP is conjugated into plastics, it can fall off plastic products and disperse in the ecosystem easily (Latini, 2005; Souza et al., 2014). Commonly, DMP can migrate into soils via irrigation and the application of pesticides and plastic films (Cartwright et al., 2000; Erythropel et al., 2012; Wang et al., 2014). Considering its mutagenicity, teratogenicity and carcinogenicity, both the United States Environmental Protection Agency and the China State Environmental Protection Administration have listed DMP as an environmental priority pollutant (Jin et al., 2013).

The black soils (Mollisols) in China are the one of three major black soil resources in the world (Liu et al., 2014a). They are extremely important for maintaining China's food security (Xu et al., 2010), because they contribute to about 14% of the total crop production and 40% of the total soybean yield in China. However, the studies showed that a serious pollution situation exists there by PAEs, and in which DMP was the most dominant one (Zhang et al., 2015).

Microbes and enzymes are the vital active components of soils. They contribute to ecosystem sustainability due to their cosmopolitan survival, massive efficient genetic pool, catabolic versatility and stress tolerance potential (Singh, 2015). Because they are highly sensitive to changes in soil conditions, they have been used to assess soil quality in contaminated soils (Jusselme et al., 2013; Yang et al., 2014). Recent work indicated that there is a predictive relationship between microbial traits and the ecosystem function of soils (Allison, 2012; Graham et al., 2014). Due to the increasing emphasis on sustainable fertility and the environmental benefits of healthy soils, it is extremely important to elucidate the effects of DMP contamination on soil microbes. However, few studies are available on the effects of DMP on black soils. This study evaluates the response of the bacterial community structure and function to DMP contamination in black soils with a microcosm model.

## Materials and methods

### Study site and soil preparation

The experiments were conducted in the greenhouses of northeast Agricultural University (Harbin, China). The time was in October, 2013, which was the season of soybean maturity. The location for the sample collection was at the Harbin Xiangfang farm (45°41′N, 126°45′E) in the black soil zone. The soil samples were collected randomly from 10 tillage layers (0–20 cm) within an area of approximately 100 m^2^. No residual DMP was detected in these samples. The soil pH was measured using a pH meter after shaking the soil water suspension (1: 2.5 w/v) for 30 min. Soil cation exchange capacity (CEC) was determined using the ammonium acetate method (Wang et al., 2008). An analysis of the total organic carbon (TOC) was performed with the Total Organic Carbon Analyzer (TOC-V, Shimadzu Labs) coupled with a Solid Sample Module (model SSM-5000, Shimadzu Labs). Total nitrogen (TN) and total phosphorus (TP) were measured by the Element Analyzer (Vario EL, Elementar Analysen systeme, Hanau, Germany). Particle size distribution was analyzed by the Sedima4-12 (Soil Particle Size Analyzer, Ecotech Ecological Technology Ltd., Beijing, China). The basic properties of the black soils used in the experiment are shown in Table 1.

**Table 1:** **Physicochemical properties of black soils obtained from northeast China**.

**Soil type**	**Textural class (USDA)**	**pH**	**TOC g/kg**	**CEC cmol(+)/kg**	**TN g/k**	**TP g/kg**	**Particle fraction (%)**		
							**Clay**	**Silt**	**Sand**
Mollisols	Silty clay loam	6.01 ± 0.04	36.79 ± 0.84	37.5 ± 1.1	2.98 ± 0.23	2.56 ± 0.14	38.1 ± 0.1	54.6 ± 0.2	7.3 ± 0.1

TOC, The total organic carbon; CEC, Cation exchange capacity; TN, Total nitrogen; TP, Total phosphorus.

### Microcosm set-up

DMP (purity greater than 99.9%) was obtained from the National Standard Material Standard Sample Information Center (Beijing, China). Acetone was used as a cosolvent and was purchased from Traditional Chinese Medicine (Beijing, China). A stock solution of DMP was prepared in acetone at a concentration of 1000 mg/L and stored in the dark at 4°C.

The microcosm model was used for the study because microcosm is a valuable tool for studying the interactions between microorganisms and their soil environments (Caracciolo et al., 2013). The soil was moistened up to 80% of its maximum field water holding capacity and preincubated at 25°C for 7 d. The stock DMP solution was added to the soil in different concentrations: Control (CK), 0 mg/kg; DMP treatment 1 (DMP1), 5 mg/kg; DMP treatment 2 (DMP2), 10 mg/kg; DMP treatment 3 (DMP3), 20 mg/kg; and DMP treatment 4 (DMP4), 40 mg/kg. The same amount of acetone solution was added to the control soils because the DMP solution was made by acetone. Then, all samples were incubated in the air for 3 h to completely volatilize the acetone. The soil samples were weighed (650 g) and placed in 1 L pots (12.2 cm height × 18 cm diameter). Three pots were prepared for each microcosm sample. Soil moisture was maintained at approximately 30% throughout the experimental period. The soils were incubated in the pots for 25 d in the dark at 25°C ± 2°C with 70 ± 5% relative humidity. Three samples were taken out from each pot, and then mix them together for homogenization using Bead beater (Bio-Spec). For the analysis of microbial community, microbial DNA was extracted from the homogenized samples, which was collected only on the 25th d post-DMP treatment, and sequenced by Illumina-MiSeq sequencing. For the analysis of microbial functional diversity, the homogenized samples were analyzed by Biolog-ECO Plates. Four time points were selected for the incubation period, they were 0, 5, 10, and 25 d, respectively. For the analysis of soil enzyme activities, the homogenized samples were used to measure the activities of urease, protease, catalase, invertase, and polyphenol oxidase, which mediate C and N cycling in soil ecosystems, at 6 time points (0, 5, 10, 15, 20, and 25 d post-DMP treatment).

### DNA extraction and amplicon generation

Total DNA from soils was extracted (0.5 g wet weight) with the E.Z.N.A. Soil DNA Kit (Omega Bio-tek, Norcross, GA, U.S.) according to the manufacturer's instructions. The extracted DNA was diluted with TE buffer (10 mM Tris-HCl and 1 mM EDTA, pH 8.0) and stored at −20°C prior to use.

The V3-V4 region of the bacterial 16S ribosomal RNA gene was amplified by PCR using the following protocol: 95°C for 2 min, followed by 25 cycles at 95°C for 30 s, 55°C for 30 s, and 72°C for 30 s, with a final extension at 72°C for 5 min. The following primers were used to attach a barcode (an eight-base sequence unique to each sample): 338F (5′-barcode-ATG CAG GGA CTA CHV GGG TWT CTA AT-3′) and 806R (5′-ACT CCT ACG GGA GGC AGC A-3′) (Kuczynski et al., 2012; Peiffer et al., 2013). PCR reactions were performed in triplicate in a 20 μL mixture containing 4 μL of 5 × FastPfu Buffer, 2 μL of 2.5 mM dNTPs, 0.8 μL of each primer (5 μM), 0.4 μL of Fast-Pfu polymerase, and 10 ng of template DNA. Negative control samples were treated similarly but excluded the template DNA; these negative controls failed to produce visible PCR products.

### Illumina-MiSeq sequencing and processing of sequencing data

After PCR process, DNA amplicons were purified with the Axy Prep DNA Gel Extraction Kit (Axygen Biosciences, Union City, CA, U.S.) and quantified using QuantiFluor™-ST (Promega, U. S.). Purified amplicons were pooled in equimolar ratios and paired-end sequenced (2 × 250) on an Illumina-MiSeq platform according to standard protocols.

Raw fastq files were demultiplexed and quality-filtered using QIIME (Quantitative Insights into Microbial Ecology, version 1.17) with the following criteria: 250 bp reads were truncated at any site receiving an average nucleotide length <20 over a 10 bp sliding window, and truncated reads shorter than 50 bp were discarded. Following exact barcode matching, sequences with 2 nucleotide mismatches in primer matching or reads containing ambiguous characters were removed. Only sequences that overlapped in regions longer than 10 bp were assembled according to their overlapping sequence. Finally, the reads that could not be assembled were discarded and the proportion of them was 0.1% out of the total reads.

To characterize the microbial diversity, operational taxonomic units (OTUs) were clustered with a 0.97 similarity cutoff using UPARSE (version 7.1 http://drive5.com/uparse/), and chimeric sequences were identified and removed using UCHIME (Quantitative Insights into Microbial Ecology) (Caporaso et al., 2010; Kunin et al., 2010).

Chao index (*S*_*Chao*1_) (the estimated number of OTUs), which refers to the species richness, was calculated using the following formula:

(1) $$S_{Chao1}=S_{obs}+\frac{n_{1}\left(n_{1}-1\right)}{2\left(n_{2}+1\right)}$$

where *S*_*obs*_ is the observed number of OTUs, *n*_1_ is the number of OTUs with only one sequence, and *n*_2_ is the number of OTUs with only two sequences (Liu et al., 2014b).

Shannon index (*H*_*Shannon*_) (Schloss et al., 2009), which indicates the community diversity within the sample (Jurasinski et al., 2009), was determined as follows:

(2) $$H_{Shannon}=-\sum_{i=1}^{S_{obs}}\frac{n_{i}}{N}ln\frac{n_{i}}{N}$$

where *S*_*obs*_ is the number of observed OTUs, *n*_*i*_ is the number of individuals in the *i*th OTU, and *N* is the total number of individuals in the community.

The phylogenetic affiliation of each 16S rRNA gene sequence was analyzed using the RDP Classifier (http://rdp.cme.msu.edu/) against the silva (SSU115) 16S rRNA database with a confidence threshold of 70% (Amato et al., 2013). Furthermore, principal co-ordinate analysis (PCoA) was conducted to estimate the β-diversity (referring to the community diversity between samples) (Lozupone and Knight, 2005) in the Illumina-MiSeq sequencing data based on the unweighted UniFrac distance matrix (Peiffer et al., 2013) created by QIIME (Caporaso et al., 2010).

### Biolog-ECO plate analysis

A total of 10 g of a fresh soil samples (soil moisture content was tested prior to weighing) were suspended in triangular flasks filled with 90 mL of sterile buffer solution (0.85% NaCl) in a biological safety cabinet. Then, the flasks were sealed and incubated in a shaker for 30 min. After settling for 10 min, the soil suspensions were obtained and diluted 1:1000 with sterile inoculating solution (0.85% NaCl). Then, 0.1 mL of each soil suspension was transferred into each of the 96 wells on the Biolog-ECO (BIOLOG, Hayward, USA) plates. The plates were incubated in the dark at 28°C, and the absorbance was read after 72 h of incubation using a micro-plate reader (BIOLOG Inc., USA) with an appropriate filter (600 nm).

The reading from each well of the Biolog-ECO plate was corrected by subtracting the value of the water blank from the replicate. Average well color development (*AWCD*), which reflects microbial activities, was determined for each plate according to the method of Garland (1997) using the following equation:

(3) $$AWCD_{ECO}=\frac{1}{31}\sum_{i=1}^{31}\left(R_{it}-R_{0t}\right)$$

where *R*_*it*_ and *R*_0*t*_ are the absorbency values of the sole carbon source *i* and the water blank at time *t.*

The metabolic functional diversity of soil microbial community can be descripted by the Shannon index *H*′, Simpson index *D* and evenness index *E* (McIntosh index) based on the absorbance reading at 72 h (Zhang et al., 2013). The equations for the calculation of each index were (Bronwyn and Raymond, 1997) as follows:

The Shannon index *H*′:

(4) $$H^{\prime }=-\sum p_{i}\times \text{ln}(p_{i})$$

where *p*_*i*_ is the proportion of the absorbance of each well to the sum of the absorbance of all wells.

The Simpson's index *D*:

(5) $$D=\;1-\sum {\left(p_{\text{i}}\right)}^{2}$$

where *p*_*i*_ is the proportion of the absorbance of each well to the sum of the absorbance of all wells.

The evenness index (McIntosh index) *E*:

(6) $$E=\frac{N-U}{N-N/\sqrt{S}}$$

(7) $$U=\sqrt{\sum n_{i}^{2}}$$

where *N* is the combined absorbance of all wells, *S* is the number of wells with color changes, and *n*_*i*_ is the absorbance of the *i*th well.

Here, index *H*′ is used to estimate the microbial diversity, index *D* is used to indicate the dominant microbial diversity, and index *E* is used to show the microbial distribution uniformity, respectively (Juliet et al., 2002; Sala et al., 2006). A total of 31 carbon sources are included in each Biolog-ECO plate and they are classified into 6 major groups (amino acids, carbohydrates, polymers, esters, carboxylic acids and phenolic compounds) (Godoi et al., 2014). The differences on utilizing the carbon sources will reflect the changes on the soil microbial communities.

### Soil enzyme assays

Urease was measured by the phenol sodium colorimetric method, and catalase was assessed by potassium permanganate titration (Yang et al., 2007). Protease was determined as reported by Ladd and Butler (Ladd and Butler, 1972). Invertase was determined using sucrose as the substrate (Guan, 1986). Polyphenol oxidase was measured according to the method of Saiya-Cork et al. (2002). All of the enzyme assays were performed in quadruplicate using two types of controls [i.e., substrate alone and soil-cocktail (everything but no substrate)].

### Statistical analyses

Software R (R Development Core Team, 2008) were used to calculate the percentage of classifiable reads and to generate the figures. Analyses of variance (ANOVA) and the least significant ranges test (Duncan's method) were performed with SPSS17.0 to test the significance (*p* < 0.05) of the differences among the treatments.

## Results

### Response of bacterial community structures and biodiversity to DMP

Illumina-MiSeq sequencing was applied to analyze the differences in microbial communities among the samples treated with DMP for 25 days. In order to analyze the microbial richness and diversity, a large number of reads (in the range of 22,825–29,011) were obtained for 16S rRNA gene amplicons of each sample, OTUs were derived, and then the *S*_*Chao*1_ and *H*_*shannon*_ indices were calculated. The data were summarized in Table 2. As it was shown, the *S*_*Chao*1_ and *H*_*shannon*_ indices of the samples were declined with increasing DMP concentration.

**Table 2:** **The comparisons of microbial community richness and α-diversity for the five DMP treatments of soil microorganisms (CK, DMP1, DMP2, DMP3, DMP4) after 25 days incubation in the dark at 25°C and 70% relative humidity**.

**Sample ID**	**Reads**	**OTUs_0.97_**	***S*_*Chao*1_**	***H*_*Shannon*_**
CK	26426 ± 3547ab	1213 ± 102a	38051 ± 1258a	5.88 ± 0.17a
DMP1	29011 ± 2581a	1210 ± 158a	31655 ± 2361b	5.69 ± 0.21ab
DMP2	24164 ± 3369ab	1175 ± 208a	30811 ± 3058b	5.62 ± 0.11b
DMP3	26788 ± 2978ab	1179 ± 340a	29538 ± 3947bc	5.53 ± 0.24bc
DMP4	22825 ± 3064b	1160 ± 147a	26332 ± 2845c	5.47 ± 0.13c

Different letters showing significant difference at p = 0.05 using Waller Duncan test.

**Figure 1:**
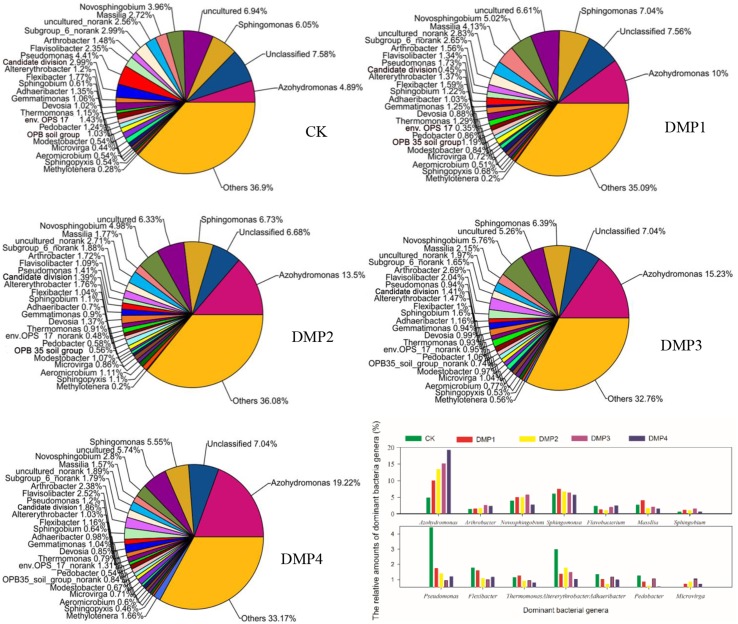
**The comparisons among the community structures and relative abundances of the microbial genera recovered from black soils and incubated for 25 days in the dark at 25°C and 70% relative humidity.** DMP concentration varied from 0 to 40 mg/kg soil (CK, DMP1, DMP2, DMP3, and DMP4).

A total of 326 genera were identified from these samples and used to study the bacterial community structures. Figure 1 shows that DMP treatment resulted in transformation of the bacterial community structure. Many bacterial genera with higher relative percentages were identified in black soils, such as *Azohydromonas* (4.89–19.22%), *Sphingomonas* (5.47–7.56%), *Novosphingobium* (2.80–5.76%), *Massilia* (1.57–4.13%), *Arthrobacter* (1.38–2.69%), *Flavisolibacter* (1.34–2.52%), *Pseudomonas* (0.94–4.41%), *Altererythrobacter* (1.03–2.99%), *Flexibacter* (1.00–1.77%), *Sphingobium* (0.61–1.60%), *Adhaeribacter* (0.70–1.35%), *Gemmatimonas* (0.90–1.25%), *Devosia* (0.85–1.37%), *Thermomonas* (0.79–1.29%), *Pedobacter* (0.54–1.24%), *Modestobacter* (0.54–1.07%), *Microvirga* (0.44–1.04%), *Aeromicrobium* (0.51–1.11%), *Sphingopyxis* (0.46–1.10%), and *Methylotenera* (0.20–1.66%). The changes on the relative percentages of *Azohydromonas* (*r*^2^ = 0.847^*^, *n* = 15), Microvirga (*r*^2^ = 0.921^**^, *n* = 15) and *Methylotenera* (*r*^2^ = 0.918^**^, *n* = 15) were significant correlated with the DMP concentration. The relative percentages of *Pseudomonas, Flexibacter, Thermomonas, Pedobacter, Flavobacterium*, and *Pontibacter* were obviously reduced as the DMP concentration increased. Interestingly, the relative percentages of *Novosphingobium*, *Massilia, Altererythrobacter, Sphingobium, Gemmatimonas, Modestobacter, Aeromicrobium*, and *Sphingopyxis* were increased by the lower DMP concentrations (DMP1, DMP2, and DMP3), but were decreased by the highest DMP concentration (DMP4).

**Figure 2:**
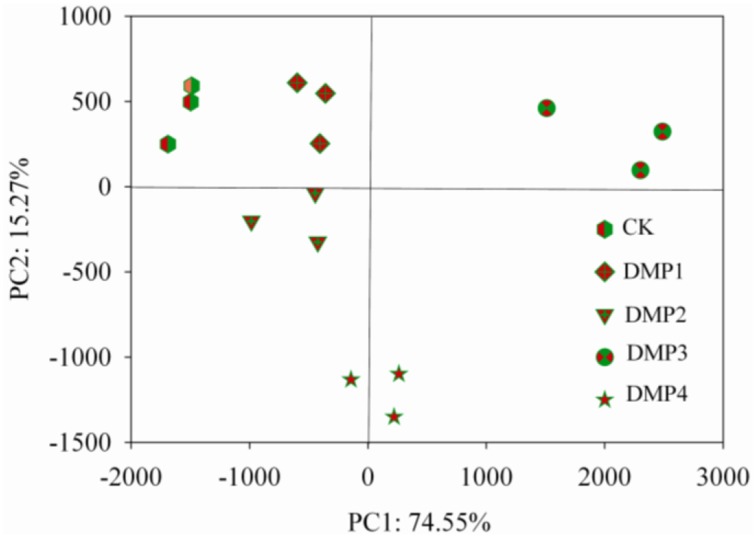
**β-diversity analysis of the microbial community across each genus by PCoA for the five DMP treatments (CK, DMP1, DMP2, DMP3, and DMP4) incubated for 25 days in the dark at 25°C and 70% relative humidity**.

The PCoA results of the bacterial β-diversity analysis covered 74.55% (PC1, *p* = 0.0011) and 15.20% (PC2, *p* = 0.0293) of the total changes of the microbial communities among the samples based on the Unweighted UniFrac distances (Figure 2). The clear changes in the β-diversity derived from this pattern occurred post-DMP exposure and increased with the increase in the DMP concentration. The PCoA revealed five separated clusters. Each cluster was distinguished due to the different DMP treatment. The analysis of β-diversity demonstrated that DMP exerted a significant effect on the community diversity.

### Response of soil microbial functions to DMP contamination

**Figure 3:**
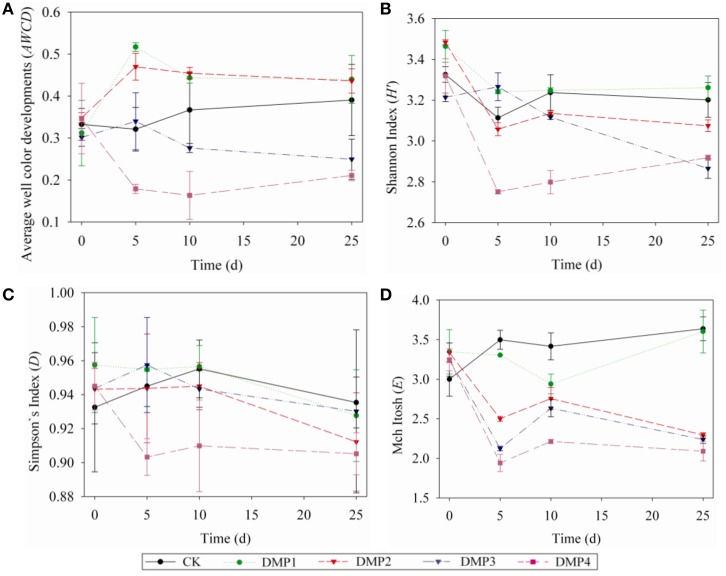
**The dynamic changes of bacterial activity and functional diversity on the microbial communities generated from the five DMP treatments (CK, DMP1, DMP2, DMP3, and DMP4) on black soil microorganisms within 25 days incubation in the dark at 25°C and 70% relative humidity. (A)** Average well color development (*AWCD*); **(B)** *H′* index (Shannon index); **(C)** *D* index (Simpson index); **(D)** *E* index (McIntosh index). The error bars indicate standard deviations (*n* = 3).

The dynamic changes on the *AWCD* (a), *H′* (b), *D* (c), and *E* (d) indices in the soils after DMP treatments were shown in Figures 3A–D. The microbial activities (*AWCD*) were increased following the treatments with low concentration (DMP1 and DMP2), however, it was significantly decreased (*p* < 0.05) following the treatments with higher DMP concentration (DMP3 and DMP4) compared to the CK (Figure 3A). The microbial diversities (*H′* indices) were not significantly changed in the treatments of DMP1 DMP2, and DMP3 compared to CK, but it was significantly reduced (*p* < 0.05) in DMP4 throughout the incubation period (Figure 3B). The dominant microbial diversities (*D* indices) were not significantly changed in DMP1 DMP2, and DMP3 compared to CK, but it was significantly reduced in DMP4 compared to CK throughout the incubation period (Figure 3C). The microbial distribution uniformities (*E* indices) were decreased (*p* < 0.05, *r*^2^ = 0.683) by the increases in the DMP concentration (Figure 3D).

**Figure 4:**
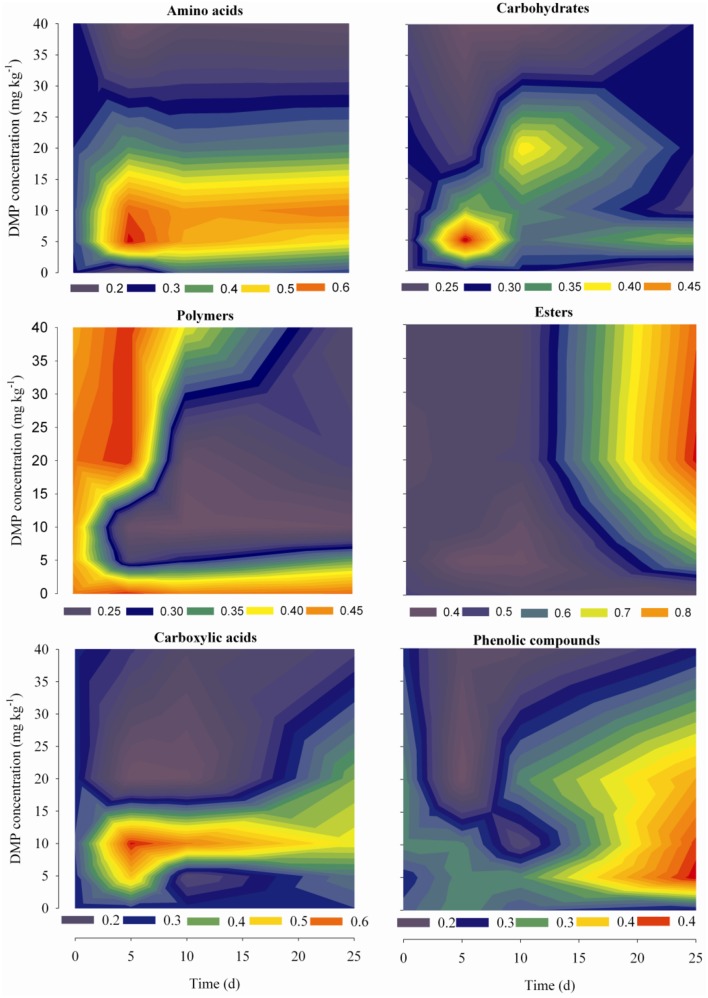
**The kinetic characteristics of carbon substrate utilization tested in Biolog-ECO plates for the five treatments (CK, DMP1, DMP2, DMP3, and DMP4) on black soil microorganisms incubated for 25 days in the dark at 25°C and 70% relative humidity**.

Dynamic analyses were performed for the microbes from the soil samples by use of 6 groups of carbon sources in the Biolog-ECO plates; the results were shown in Figure 4. The remarkable differences were observed by comparing the profiles of carbon utilization among the soil samples. The utilization of amino acids and carboxylic acids were significantly promoted by low DMP concentration (DMP1 and DMP2), and significantly inhibited by higher DMP concentration (DMP3 and DMP4) throughout the incubation period. The utilization of carbohydrates and phenolic compounds was promoted by low DMP concentration (DMP1, DMP2, and DMP3), and inhibited by DMP4 throughout the incubation period. The utilization of polymers was inhibited by low DMP concentration (DMP1 and DMP2), and promoted by higher DMP concentration (DMP3 and DMP4) at the beginning, and inhibited later. The utilization of ester was promoted by DMP treatments in a dosage-dependent manner, that is, it was correlated positively (*r*^2^ = 0.759^*^, *n* = 20) with the DMP concentration (Figure 4).

### Response of soil enzymes to DMP contamination

**Figure 5:**
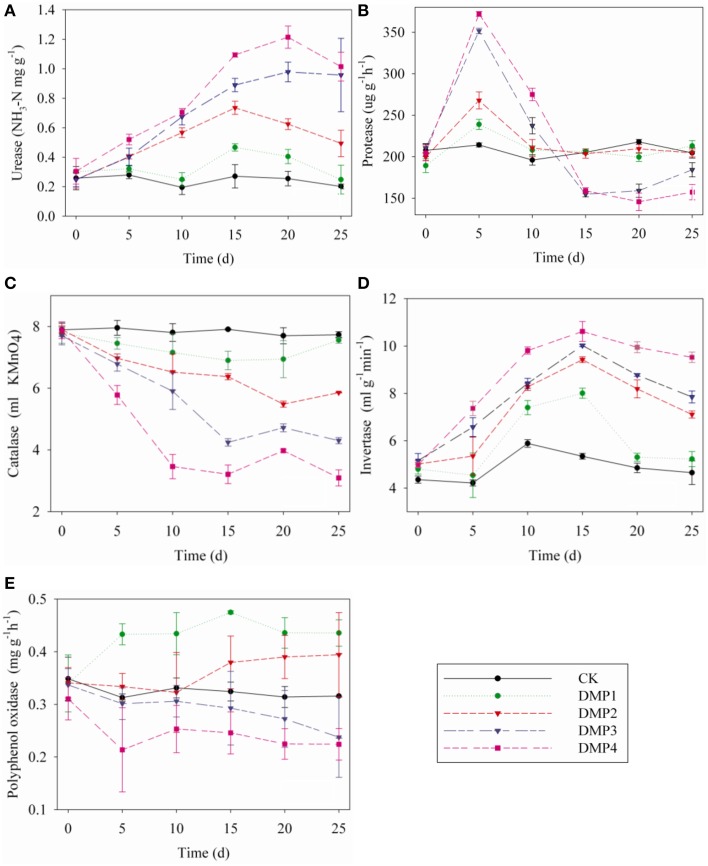
**Effects of DMP contamination on the enzyme activities from black soil microorganisms with 25 days incubation in the dark at 25°C and 70% relative humidity.** DMP concentration varied from 0 to 40 mg/kg soil (CK, DMP1, DMP2, DMP3, and DMP4). **(A)** Urease; **(B)** Protease; **(C)** Catalase; **(D)** Invertase; **(E)** Polyphenol Oxidase. The error bars indicate standard deviations (*n* = 3).

The dynamic changes in soil enzymes caused by DMP contamination throughout the incubation period were shown in Figures 5A–E. DMP significantly increased the activities of urease and invertase (*r*^2^ = 0.67^*^ and *r*^2^ = 0.59^*^) within 25 days (Figures 5A,D). Conversely, the catalase activity was significantly inhibited (*r*^2^ = 0.75^*^) by the increased DMP concentration (Figure 5C). Protease activity was increased by DMP at the beginning, but decreased in higher DMP concentration (DMP3 and DMP4) later (Figure 5B). Polyphenol oxidase activity was promoted by low DMP concentration (DMP1 and DMP2), but significantly inhibited by higher DMP concentration (DMP3 and DMP4) throughout the incubation period (Figure 5E).

## Discussion

Microbes are the most important components of soils, and are a useful measure for soil health status and ecology. Illumina-MiSeq sequencing provides an unprecedented power for the deep understanding of bacterial community composition and diversity based on 16S rRNA gene libraries (de Gannes et al., 2013; Peiffer et al., 2013). Hence, the method was applied to analyze differences on microbial communities among the samples in this study. The results showed that the OTU richness and microbe diversity in black soils were gradually reduced by increasing DMP concentrations (Table 2). The results of this study basically correspond with the results obtained previously by Zhang et al. (2010), however, who only found polycyclic aromatic hydrocarbons (PAH) decreased the microbial richness and diversity in soil. But the effects of DMP were opposite to the effects of dioxins on soil microbial richness and diversity in (Hanano et al., 2014).

Additionally, it was observed that DMP treatments resulted in transformation of the microbial community (Figures 1, 2). All of the bacterial communities in the black soils were dominated by 21 major genera (the relative percentage must be >0.2%). Although the total numbers of the major genera were not changed, but the relative percentages of the genera were altered by DMP contamination (Figure 1). *Azohydromonas* and *Microvirga* are key players in the nitrogen cycle in soils (Coelho et al., 2008; Ardley, 2012; Yousuf et al., 2014), and the relative percentages of *Azohydromonas* increased along with the increase in DMP concentration. The relative percentages of *Microvirga* increased along with the increase in lower DMP concentration, but dropped in the highest DMP concentration. The reason for the drop cannot be found at present. The relative percentages of *Methylotenera* increased along with the increase in DMP concentration, but the functions in soils for this bacterium are not clear. However, some genera that were distributed broadly in soils and have been shown to be beneficial to soil health were inhibited by increasing DMP concentration (Figure 1). These genera included *Pseudomonas* (Troxler et al., 2012; Dharni et al., 2014; Stelting et al., 2014), *Flexibacter* (Jones and Knowles, 1991; Drijber and McGill, 1994), *Devosia* (Rivas et al., 2002; Ryu et al., 2008; Hassan et al., 2014), and *Flavobacterium* (Das et al., 2012; Kolton et al., 2014). The relative percentages of some genera increased by the lower DMP concentrations (5, 10, and 20 mg/kg), but then they were inhibited by the high DMP concentration (40 mg/kg), such as the case in *Microvirga* (Figure 1). The genera observed to follow this trend showed to have a capability to degrade organic pollutants in soils, such as *Novosphingobium* (Yan et al., 2007; Saxena et al., 2013), *Massilia* (Faramarzi et al., 2009), *Altererythrobacter* (Dunlevy et al., 2013), *Sphingobium* (Cunliffel and Kertesz, 2006; Raina et al., 2008), and *Sphingopyxis* (Jindal et al., 2013). The results indicated DMP contamination could stimulate some genera that have resistance of DMP, and inhibit some genera that are damaged by DMP. The analysis by PCoA indicated that β–diversity was changed by DMP treatments (Figure 2). This may be due to the accumulation of DMP in the hydrophobic regions of the microbial membrane and the disruption of membrane fluidity (Cartwright et al., 2000), and finally inhibit the growth of some microbes.

The changes on the soil microbial community also were supported by the study of Biolog-Eco plate. The *AWCD* value from the study of Biolog-Eco plate is commonly used as an indicator of the total metabolic activity of soil microbes (Wu et al., 2014). In this study, compared to the control, the *AWCD* values were increased in soils treated with low DMP concentration (DMP1 and DMP2), but were significantly decreased in soils treated with higher DMP concentration (DMP3 and DMP4) throughout the incubation period. This meant that the total metabolic activity was changed, and indirectly indicated that the soil microbial community was changed by DMP. The functional diversity indices (*H′*, *D*, and *E*) of soil microbes can be used to estimate the microbial diversity, dominant microbial diversity and microbial distribution uniformity, respectively. In this study, the microbial distribution uniformity was reduced by DMP with all concentration (DMP 1, DMP2, DMP3, and DMP4), and also the microbial diversity was reduced by higher concentrations (DMP2, DMP3, and DMP4). The dominant microbial diversity appears to have significantly decreased due to the treatment DMP4 compared to CK throughout the incubation period. Similar to our results, Fang et al. (2015) reported changes in the *AWCD* and diversity indices in atrazine-treated soil. The utilization for different carbon sources reflect metabolic features of soil microbes (Wang et al., 2009). The changes on the utilization of different carbon sources reflect the changes on soil microbial community (Kong et al., 2013; Zhang et al., 2013). The changes on the utilization of different carbon sources (Figure 4) indicated that the metabolic functions of soil microbes were sensitive to DMP contamination, and the metabolic features of soil microbes were changed, which coincided with the results from the *AWCD* and biodiversity assessments (Figure 3).

Soil enzymes are involved in biological cycling and the development of fertility; thus, they are crucial indicators of soil biochemistry. The activities of soil enzymes are used to estimate the adverse effects of various pollutants on soil health (Masto et al., 2008; Sun et al., 2014) because they are easily affected by the physical, chemical and biological factors of soils (Ma et al., 2015). In this study, the activities of five enzymes were tested, all of them were altered after the treatment of DMP (Figure 5). The activities of catalase, polyphenol oxidase and invertase may reflect the conversion rate of soil organic carbon, while the activities of urease and protease may reflect soil nitrogen metabolism (Schloter et al., 2003; Kaplan et al., 2014). The results of soil enzymes suggested that DMP pollution could alter the metabolism of carbon and nitrogen in black soils. These findings were similar to the Illumina-MiSeq sequencing data and the biology-ECO plate analysis results. Therefore, DMP treatments altered the bacterial community and functions in black soils.

## Conclusions

To ensure agricultural sustainability and the normal functioning of soils, it is necessary to maintain the viability and diversity of soil microbe populations. DMP contamination decreased bacterial richness and diversity, changed the bacterial community structure, increased the number of some genera associated with nitrogen metabolism, inhibited the number of other genera that were extremely beneficial to soil health, and disturbed the activities of some enzymes and metabolic functions of the microbes in black soils. These findings propose that DMP causes a direct impact on ecosystem function of black soils.

### Conflict of interest statement

The author declares that the research was conducted in the absence of any commercial or financial relationships that could be construed as a potential conflict of interest.
