# Supplementary material for: Impacts of dimethyl phthalate on the bacterial community and functions in black soils
Source: Front Microbiol. 2015 May 5;6:405. doi: 10.3389/fmicb.2015.00405 (PMC4419729; doi:10.3389/fmicb.2015.00405)
Supplement: Supplementary file 1 [file DataSheet1.DOCX]

Supplementary data for the three genera (*Azohydromonas, Methylotenera* and *Microvirga*)
